# TRANSITION INTO RECEIVING CARE AND DEPRESSIVE SYMPTOMS AMONG KOREAN OLDER ADULTS

**DOI:** 10.1093/geroni/igae098.3758

**Published:** 2024-12-31

**Authors:** Haeun Yoo, Kyungmin Kim

**Affiliations:** Seoul National University, Seoul, Republic of Korea; Seoul National University, Seoul, Republic of Korea

## Abstract

Although transitioning into receiving care in late life is common, its psychological impact remains underexplored. Adaptation theory suggests that depressive symptoms may peak when individuals start receiving care but diminish over time as they adapt. On the other hand, they may become more depressed over time as their health conditions decline. Furthermore, the availability of a larger care resource network, including formal and informal caregivers, may buffer the negative impact of receiving care on depressive symptoms. This study aims to examine how transitioning into receiving care is associated with changes in depressive symptoms, and how the association varies by perceived care resources. Utilizing panel data from the Korean Longitudinal Study of Ageing (biennial surveys; 2008–2022), we compared two groups of older adults (aged 65–105; observation N = 3,739), based on care status at two consecutive waves: (a) received care at both waves and (b) newly received care at later wave. We found that older adults who transitioned into receiving care showed greater increases in depressive symptoms than those who continued receiving care, adjusting for participants’ sociodemographic/health characteristics as well as depressive symptoms at prior wave. Moreover, the association between transitioning into receiving care and changes in depressive symptoms differed depending on perceived care resources; thus, the negative psychological impact of transitioning into receiving care was significant only for participants with fewer perceived care resources at prior wave. The findings highlight the importance of a well-established support network in the context of disability.

